# Polysubstance use disorders among US adults

**DOI:** 10.1038/s41380-026-03618-z

**Published:** 2026-04-29

**Authors:** Beth Han, Nora D. Volkow, Christopher M. Jones, Deborah Dowell, Grant Baldwin, Emily B. Einstein, Geetha A. Subramaniam, Yngvild Olsen, Carlos Blanco, Wilson M. Compton

**Affiliations:** 1 National Institute on Drug Abuse, National Institutes of Health, Bethesda, MD US; 2 Substance Abuse and Mental Health Services Administration, Rockville, MD US; 3 Centers for Disease Control and Prevention, Atlanta, GA US

**Keywords:** Addiction, Neuroscience

## Abstract

Polysubstance use disorders ( ≥ 2 substance use disorders (SUDs)) are associated with high morbidity and mortality. We analyzed data from 92,233 adult participants in the 2022–2023 US National Surveys on Drug Use and Health to estimate past-year prevalence of polysubstance use disorders and to examine their associations with age of substance use initiation. Multivariable logistic regression and Poisson regression were applied. Age- and sex-adjusted past-year prevalence of 2 SUDs was 19.2–44.9% (95% CIs=11.1–62.3%) among adults with any SUD. Age- and sex-adjusted past-year prevalence of ≥3 SUDs ranged from 16.4% (95% CI = 14.3–18.6%) among adults with cannabis use disorder, to 32.4–44.7% (95% CIs=29.1–51.3%) among those with opioid use disorder or prescription stimulant or tranquilizer/sedative use disorder, and up to 48.2–72.0% (95% CIs=39.4–81.7%) among those with methamphetamine, cocaine, or hallucinogen use disorder. Overall, compared to adults who initiated substance use before age 18, the number of SUDs was 73–83% lower for those who initiated at age ≥21 (range of incidence density ratios (IDRs)=0.17–0.27, 95% CIs=0.12–0.31). Specifically, compared with corresponding adult counterparts who initiated before age 18, the number of moderate-severe SUDs was 32% lower among those initiating alcohol at ages 21–29 (IDR = 0.68, 95% CI = 0.57–0.83), 21% lower among those initiating cannabis at ages 21–29 (IDR = 0.79, 95% CI = 0.69–0.90), and 45–62% lower (IDRs=0.38–0.55, 95% CIs=0.31–0.76) among adults who never initiated alcohol, cannabis, or nicotine use. The elevated prevalence of polysubstance use disorders associated with early initiation of substance use underscores the critical need for evidence-based strategies to prevent alcohol, cannabis, and nicotine consumption before age 21.

## Introduction

Polysubstance use disorders (having ≥2 substance use disorders (SUDs)) are associated with significant morbidity, poor treatment adherence and outcomes, and high mortality [1–7]. Polysubstance use disorders reflect shared neurobiological mechanisms, with addictive drugs inducing common mesolimbic dopamine adaptations that strengthen reinforcement and promote cross‑sensitization across substances [1, 8]. Moreover, shared developmental [8–11] and genetic [2, 12, 13] vulnerabilities interact with environmental factors [14, 15], collectively increasing risk across drug classes and sustaining persistent polysubstance use and use disorders.

However, significant gaps persist in our understanding of the current prevalence, severity, and correlates of polysubstance use disorders. Existing studies’ reliance on data collected more than a decade ago, the exclusion of nicotine dependence, or a narrow focus on opioid misuse and opioid use disorder, constrain our understanding of current polysubstance use disorders and hinder the development of effective prevention and treatment strategies.

In particular, the epidemiology of SUDs has evolved since researchers examined national data from 2012–2013 [16]. While alcohol use disorder declined during 2002–2019, drug use disorders (e.g., cannabis and methamphetamine use disorders) increased during 2015–2019 [17–19]. Few studies of polysubstance use disorders include nicotine [7, 16, 20], despite its widespread use among adults, common co‑use with alcohol, cannabis, and other drugs [3, 21–23], and links between nicotine dependence and persistent co‑occurring SUDs due to shared neurobiological pathways, notably dopaminergic enhancement and nicotine’s potentiating effects of other substances [21, 24–30].

National data from 2012–2013 revealed that 93.3% of adults with opioid use disorder used ≥2 substances and 26.1% met criteria for polysubstance use disorder [20]. Similar polydrug use patterns were reported from 2017–2019 data among people misusing prescription opioids and those using heroin [31]. None of the studies examined prescription opioid, stimulant, and sedative/tranquilizer use disorders among people reporting no misuse of these medications. Yet, “medically guided adults” represent a significant polysubstance use group that warrants clinical attention and inclusion in research on polysubstance use disorders [32–34].

Assessing how the age of onset relates to polysubstance use disorders is also directly relevant to prevention and intervention strategies. In addition to sociodemographic and mental health correlates of polysubstance use [6, 20] and use disorders [7, 16], early substance use during a period of neurodevelopmental immaturity in the reward, executive‑control, and stress‑regulation circuits heightens sensitivity to addictive substances and increases vulnerability to polysubstance use and use disorders [35, 36]. Early initiation can disrupt the development of cognitive control, emotional regulation, and decision‑making, creating a cascade that elevates the risk of polysubstance use [36, 37]. Early exposure also strengthens associative learning—such as conditioned cues, expectancies, and cross‑sensitization—facilitating progression from single‑ to polysubstance use [38]. Notably, early use often occurs in peer or family environments that reinforce substance‑use behaviors, further promoting polysubstance use [39]. However, no study has examined the relationships between the age of overall and specific substance use initiation and polysubstance use disorders, using recent nationally representative US data and assessing specific SUDs based on DSM-5 diagnostic criteria. To inform clinical practice and policy, it is essential to understand the relationships between age of initiation—both overall and substance-specific—and moderate-severe polysubstance use disorders.

Using 2022–2023 nationally representative data, we examined the following questions and our hypothesis:

- What is the national prevalence of overall and moderate-severe polysubstance use disorders? How does the prevalence vary by age of initiation of overall and specific substance use? What is the national prevalence of specific combinations of polysubstance use disorders?
- How is the age of initiation—both overall and substance-specific—associated with overall and moderate-severe polysubstance use disorders? We hypothesized that adults who initiated substance use before age 18 would be more likely to have overall and moderate-severe polysubstance use disorders, compared with those who initiated at an older age.

## Methods

### Data sources

We examined nationally representative data from 92,233 US civilian, noninstitutionalized adults aged ≥18 who participated in the 2022–2023 National Surveys on Drug Use and Health (NSDUH) [6, 34, 35]. The Institutional Review Board at the Research Triangle Institute International approved the NSDUH data collection protocol. NSDUH used multimode (in-person/online) data collection [33, 34, 40, 41]. Each participant provided informed consent [40, 41]. The mean NSDUH weighted household screening response rate was 25.0%, and the mean NSDUH weighted interview response rate for adults was 49.5% [40, 41].

### Measures

NSDUH collected past-year use of tobacco products or nicotine vaping, alcohol, cannabis, cocaine, heroin, hallucinogens, methamphetamine, inhalants, and illegally made fentanyl, past-year use and misuse of psychotropic medications (prescription opioids, stimulants, and sedatives/tranquilizers), and age of specific substance use initiation [40, 41]. To reduce recall bias, NSDUH did not collect the age of misuse initiation for respondents who began misusing psychotropic medications prior to the past year.

Psychotropic use included using one’s own prescription as directed by a doctor as well as misuse [33, 34, 40, 41]. NSDUH classified misuse of psychotropic medications when respondents endorsed any of the statements describing their use at any point in the past year: “without a prescription of my own”; “in greater amounts than prescribed”; “more often than prescribed”; “longer than prescribed”; or “in some other way a doctor did not direct me to use” [33, 34, 40, 41]. Opioid misuse was defined as misuse of prescription opioids or use of heroin or illegally made fentanyl.

Using DSM-5 diagnostic criteria, NSDUH assessed past-year alcohol and other specific drug use disorders and their severity (moderate-severe: having ≥4 DSM-5 symptoms) and major depressive episode (MDE) [40, 41]. The 2022–2023 NSDUH assessed prescription opioid, stimulant, and sedative/tranquilizer use disorders among people who used these medications regardless of their misuse status [33, 34, 40, 41]. NSDUH examined past-month nicotine dependence using the Nicotine Dependence Syndrome Scale and Fagerstrom Test of Nicotine Dependence [40–42]. Additionally, NSDUH assessed sociodemographic characteristics, self-rated health, past-year emergency department visits, and past-year suicidal ideation [40–42].

### Statistical analyses

We conducted separate (i.e., independent), multivariable, multinomial logistic regression analyses to estimate the age- and sex-adjusted prevalence of the number of different substances used/misused in the past year and the number of SUDs (1) among adults with past-year use/misuse of a specific substance and (2) among adults with a specific past-year SUD. Similarly, we also estimated the number of moderate-severe SUDs among adults with a specific past-year moderate-severe alcohol or drug use disorder, after controlling for age and sex. We conducted separate (i.e., independent), multinomial logistic regression analyses to estimate the prevalence of the number of substances used/misused in the past year and the number of SUDs overall and stratified by age of initiation of overall and specific substance use among adults.

We provide a more detailed breakdown of the prevalence of the number of SUDs, including the prevalence of having 1 SUD, 2 SUDs, and ≥3 SUDs, helping to understand the detailed scope of substance use disorders and quantify their impacts more precisely. The prevalence of ≥2 SUDs is the sum of the prevalence of 2 SUDs and the prevalence of ≥3 SUDs.

We estimated prevalence of specific combinations of SUDs overall and by age and sex. Multivariable Poisson regressions [43, 44] were applied to examine associations between age of initiation of overall and substance-specific use and count outcomes:(1) the number of SUDs (primary outcome); (2) the number of different substances used or misused in the past year (secondary outcome); and (3) the number of moderate-severe SUDs (secondary outcome) among adults after controlling for sociodemographic characteristics, health status, and mental health conditions. We examined three highly correlated outcomes; given their conceptual relatedness and correlation, we did not apply formal multiple-comparison correction but interpreted findings (especially those related to secondary outcomes) in light of potential multiplicity.

All analyses used SUDAAN software (Release 11.0.3) to account for NSDUH’s complex sample design and sample weights. For each analysis, *P* < 0.05 (2-tailed) was considered statistically significant. The STROBE reporting guideline was followed for cross-sectional studies.

## Results

### Age- and sex-adjusted past-year prevalence of overall and moderate-severe polysubstance use disorders

**Fig. 1:**
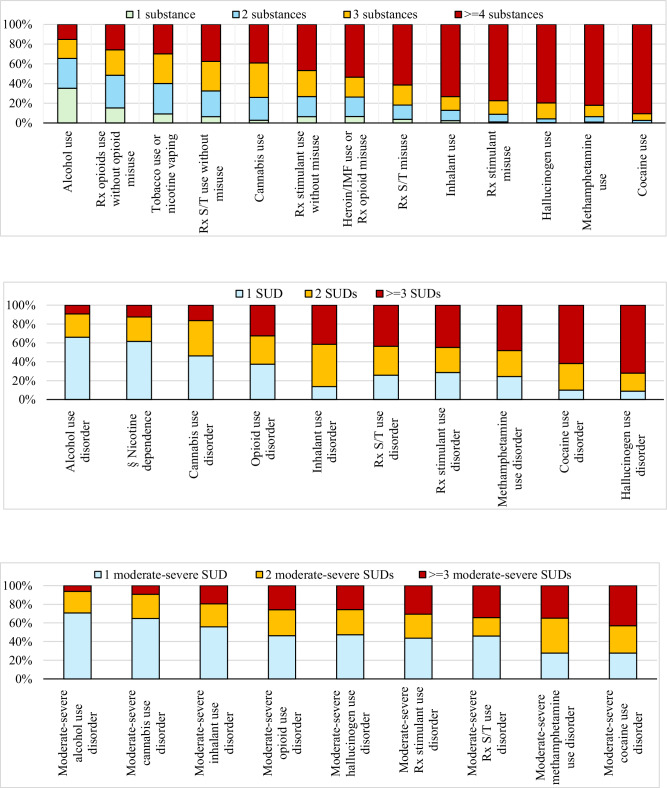
Proportions of past-year use of different substances and polysubstance use disorders in US adults. **a** Age- and sex-adjusted proportions of the number of different substances used in adults with substance use, by substance (ordered by proportion of using ≥4 substances). **b** Age- and sex-adjusted proportions of the number of substance use disorders (SUDs) among adults with a specific SUD, by SUD (ordered by proportion of having ≥3 SUDs). **c** Age- and sex-adjusted proportions of the number of moderate-severe SUDs in adults with a specific moderate-severe SUD, by moderate-severe SUD (ordered by proportion of having ≥3 moderate-severe SUDs). Figure footnote: Source: 2022-2023 National Surveys on Drug Use and Health data. Rx=prescription; Opioid misuse=Heroin, illegally made fentanyl (IMF) use, or Rx opioid misuse; S/T sedative or tranquilizer; § Past-month measure.

Over two-thirds of adults used alcohol in the past year (Supplementary Table 1). Among them, age- and sex-adjusted past-year prevalence of using ≥4 substances was 15.3% (95% CI = 14.7–15.8%) (Table 1, Fig. 1a). By contrast, past-year prevalence of opioid misuse among adults was 3.3% (95% CI = 3.1–3.6%); yet among them, age- and sex-adjusted prevalence of using ≥4 substances was 53.4% (95% CI = 51.0–55.9%). Similarly, past-year prevalence of using hallucinogens, cocaine, methamphetamine, or inhalants among adults was low (range=0.7–3.2%, 95% CIs=0.6–3.4%); but among them, the vast majority used ≥4 substances (age- and sex-adjusted prevalence range=73.2–90.5%, 95% CIs=67.3–92.8%).

**Table 1:** Age- and sex-adjusted prevalence of the number of different substances used or misused in the past year and the number of substance use disorders (SUDs) among US adults by substance and overall and moderate-severe SUD, weighted percentage (95% CI) (N = 92,233).

Substance use^a^ among adults with past-year use or misuse of a specific substance	1 substance	2 substances	3 substances	≥4 substances
Alcohol	35.3 (34.7–35.9)	30.2 (29.6–30.9)	19.3 (18.7–19.9)	15.3 (14.7–15.8)
Tobacco products or nicotine vaping	9.2 (8.6–9.9)	30.9 (29.8–32.0)	30.1 (29.1–31.1)	29.8 (28.8–30.8)
Cannabis	2.8 (2.4–3.4)	23.2 (22.0–24.4)	34.9 (33.6–36.2)	39.1 (37.9–40.4)
Rx opioids (no opioid misuse)	15.3 (14.4–16.3)	33.0 (31.9–34.2)	25.9 (24.8–27.1)	25.7 (24.9–26.6)
Opioid misuse	6.6 (5.2–8.3)	19.8 (17.5-22.3)	20.2 (18.2–22.3)	53.4 (51.0–55.9)
Rx S/T (no misuse)	6.4 (5.7–7.2)	26.2 (24.6–27.9)	29.9 (28.4–31.4)	37.5 (35.9–39.1)
Rx S/T misuse	3.8 (1.8–7.8)	14.4 (11.5–17.9)	20.4 (16.9–24.5)	61.4 (56.5–66.0)
Rx stimulants (no misuse)	6.4 (5.6–7.4)	20.4 (18.6–22.4)	26.5 (24.4–28.6)	46.7 (44.7–48.7)
Rx stimulant misuse	1.1 (0.5–2.4)	7.8 (5.9–10.4)	13.6 (11.4–16.2)	77.5 (74.0–80.6)
Hallucinogens	0.5 (0.2–1.4)	3.7 (2.4–5.6)	16.2 (13.8–19.0)	79.5 (76.6–82.2)
Cocaine	0.0 (0.0–0.1)	2.6 (1.6–4.4)	6.8 (4.7–9.8)	90.5 (87.7–92.8)
Methamphetamine	1.0 (0.4–2.9)	5.5 (3.2–9.2)	11.5 (7.6–17.2)	82.0 (76.2–86.6)
Inhalants	2.3 (1.1–4.7)	10.6 (7.2–15.3)	13.9 (10.0–19.0)	73.2 (67.3–78.4)

SUDs among adults with a specific past-year SUD, by SUD	1 SUD	2 SUDs	≥3 SUDs
Alcohol use disorder	66.1 (64.3–67.9)	24.7 (22.9–26.6)	9.2 (8.3–10.3)
Nicotine dependence	61.7 (59.7–63.6)	25.9 (24.2–27.7)	12.4 (11.2–13.8)
Cannabis use disorder	46.3 (43.4–49.3)	37.3 (34.2–40.6)	16.4 (14.3–18.6)
Opioid use disorder	37.5 (33.8–41.4)	30.1 (26.6–33.9)	32.4 (29.1–35.8)
Rx stimulant use disorder	28.7 (23.1–35.1)	26.6 (22.0–31.8)	44.7 (38.2–51.3)
Rx S/T use disorder	25.9 (19.8–33.2)	30.6 (25.4–36.4)	43.5 (36.4–50.9)
Hallucinogen use disorder	8.8 (4.2–17.5)	19.2 (11.1–31.0)	72.0 (59.7–81.7)
Cocaine use disorder	9.9 (6.4–14.9)	28.3 (21.7–36.0)	61.8 (54.3–68,8)
Methamphetamine use disorder	24.3 (17.7–32.5)	27.5 (20.9–35.4)	48.2 (39.4–57.0)
Inhalant use disorder	13.7 (5.1–37.1)^b^	44.9 (28.7–62.3)^b^	41.4 (26.9–57.7)^b^

Moderate-severe SUDs among adults with a specific past-year moderate-severe SUD, by moderate-severe SUD	1 moderate-severe SUD	2 moderatesevere SUDs	≥3 moderate–severe SUDs
Moderate-severe alcohol use disorder	70.6 (68.3–72.7)	23.2 (20.9–25.7)	6.2 (5.3–7.3)
Moderate-severe cannabis use disorder	64.7 (62.7–66.8)	26.1 (24.1–28.2)	9.2 (7.7–10.8)
Moderate-severe opioid use disorder	46.3 (41.3–51.4)	27.8 (23.7–32.4)	25.9 (21.5–30.8)
Moderate-severe Rx stimulant use disorder	43.7 (36.1–51.6)	25.7 (19.8–32.7)	30.6 (24.3–37.8)
Moderate-severe Rx S/T use disorder	46.0 (38.7–53.6)	19.7 (14.9–25.4)	34.3 (27.2–42.2)
Moderate-severe hallucinogen use disorder	47.4 (31.9–63.4)	27.0 (16.5–40.8)	25.6 (16.8–37.0)
Moderate-severe cocaine use disorder	27.7 (21.2–35.4)	29.2 (22.1–37.6)	43.1 (33.8–52.8)
Moderate-severe methamphetamine use disorder	27.6 (21.1–35.2)	37.4 (31.3–43.9)	35.0 (27.2–43.8)
Moderate-severe inhalant use disorder	55.8 (37.2–73.0)^b^	24.7 (11.0–46.6)^b^	19.5 (11.4–31.4)^b^

Data sources: 2022-2023 National Surveys on Drug Use and Health.

Rx=prescription, *S/T* sedative/tranquilizer, *Opioid misuse* misuse of Rx opioids or use of heroin or illegally made fentanyl.

^a^Different substances used/misused in the past year include alcohol, tobacco products/nicotine vaping, cannabis, opioids (prescription (Rx) opioids, heroin, or illegally made fentanyl), Rx stimulants, Rx sedatives or tranquilizers, hallucinogens, cocaine, methamphetamine, or inhalants. Substance use disorders include past-year alcohol use disorder, cannabis use disorder, opioid use disorder, Rx stimulant use disorder, Rx sedative or tranquilizer use disorder, cocaine use disorder, hallucinogen use disorder, methamphetamine use disorder, inhalant use disorder or past-month nicotine dependence.

^b^Caution is warranted when interpreting this estimate due to its low statistical precision.

Although 66.1% of adults with alcohol use disorder and 61.7% of adults with nicotine dependence did not have other SUDs, most adults with other specific drug use disorders had polysubstance use disorders (Fig. 1b, Table 1, Supplementary Table 1). Age- and sex-adjusted past-year prevalence of 2 SUDs was 19.2–44.9% (95% CIs=11.1–62.3%) among adults with any SUD; prevalence of ≥3 SUDs ranged from 16.4% (95% CI = 14.3–18.6%) among those with cannabis use disorder, 32.4–44.7% (95% CIs=29.1–51.3%) among those with opioid use disorder or prescription stimulant or tranquilizer/sedative use disorder, to 48.2–72.0% (95% CIs=39.4–81.7%) among those with methamphetamine, cocaine, or hallucinogen use disorder.

Similarly, although most adults with moderate-severe alcohol, cannabis, or inhalant use disorder did not have other moderate-severe use disorders, most adults with other specific moderate-severe drug use disorders had ≥2 moderate-severe polysubstance use disorders (Fig. 1c, Table 1, Supplemental Figure). Age- and sex-adjusted past-year prevalence of ≥3 moderate-severe SUDs ranged from 25.6–25.9% (95% CIs=16.8–37.0%) among those with moderate-severe opioid or hallucinogen use disorder to 30.6–43.1% (95% CIs=24.3–52.8%) among those with moderate-severe prescription stimulant, prescription sedative/tranquilizer, methamphetamine, or cocaine use disorder.

### Prevalence of specific combinations of polysubstance use disorders

Among US adults in the past year, 76.2% (95% CI = 75.7–76.6%) had no SUD, 17.6% (95% CI = 17.2–18.0%) had 1 SUD, 4.6% (95% CI = 4.4–4.9%) had 2 SUDs, and 1.6% (95% CI = 1.5–1.8%) had ≥3 SUDs (Supplementary Table 2). Specifically, there were 299 unique, exclusive combinations of SUDs, and prevalence of specific combinations of polysubstance use disorders varied by age and sex. For example, past-year prevalence of having alcohol and cannabis use disorders alone was 9.5 times lower among adults aged ≥50 (0.4%, 95% CI = 0.2–0.6%) than those aged 18–29 (3.8%, 95% CI = 3.5–4.1%), but it was 1.6 times higher among males (1.8%, 95% CI = 1.6–2.0%) than females (1.1%, 95% CI = 1.0–1.3%).

### The number of substances used/misused by age of initiation of substance use and never use

Past-year prevalence of using ≥4 substances was 16.9% (95% CI = 16.3–17.5%) among adults who initiated using any substance before age 18, compared with 2.3% (95% CI = 1.9–2.8%) among those who initiated it at ages 21–29 (Table 2). Specifically, past-year prevalence of using ≥2 substances was 66.8% among those who initiated alcohol use before age 18, compared with 9.8% among those who never used it. It was 67.7% among those who initiated use of tobacco products or nicotine vaping before age 18, compared with 22.9% among those who never used them; and it was 78.1% among those who initiated cannabis use before 18, compared with 26.9% among those who never used it. These results are highly consistent with those after age- and sex-adjustment (results available upon request).

**Table 2:** Prevalence of the number of different substances used or misused in the past year among adults in the US overall and by age initiation of overall and specific substance use, weighted percentage (95% CI) (N = 92,233).

Substance Use^a^	0 substance	1 substance	2 substances	3 substances	≥4 substances
Overall	17.7 (17.1–18.3)	32.3 (31.8–32.8)	24.3 (23.8–24.9)	14.5 (14.0–14.9)	11.2 (10.8–11.5)

Age of First Use of Any Substance^c^					
Age of first use of any substance					
≤17^b^	7.4 (6.9–7.9)	28.3 (27.7–29.0)	27.9 (27.2–28.6)	19.5 (18.9–20.1)	16.9 (16.3–17.5)
18–20	**11.0** (**10.0**–**12.0)**	**42.3** (**41.1**–**43.5)**	27.9 (26.8–29.1)	**12.7** (**11.7**–**13.8)**	**6.1** (**5.6**–**6.7)**
21–29	**17.5** (**16.2**–**18.9)**	**51.0** (**49.1**–**52.9)**	**22.2** (**20.7**–**23.7)**	**7.0** (**6.1**–**8.0)**	**2.3** (**1.9**–**2.8)**
≥30	**27.0** (**21.9**–**32.8)**	**45.7** (**39.8**–**51.6**)	**21.4** (**16.8**–**26.7)**	**4.6** (**3.0**–**6.9)**	**1.4 (0.6**–**3.3)**

Age of First Substance Use by Substance^c^					
Alcohol					
≤17^b^	5.7 (5.2–6.2)	27.5 (26.7–28.2)	27.7 (27.0–28.5)	20.4 (19.8–21.1)	18.7 (17.9–19.5)
18-20	**9.0** (**8.3**–**9.8)**	**39.3** (**38.2**–**40.4)**	28.8 (27.5–30.0)	**14.7** (**13.7**–**15.7)**	**8.3** (**7.7**–**9.0)**
21–29	**14.9** (**13.9**–**16.0)**	**44.0** (**42.5**–**45.4)**	**25.0** (**23.9**–**26.1)**	**11.0** (**10.1**–**12.0)**	**5.2** (**4.6**–**5.8)**
≥30	**22.1** (**17.7**–**27.3)**	**45.5** (**39.8**–**51.3)**	**23.2** (**19.8**–**26.9)**	**7.6** (**5.5**–**10.3)**	**1.7** (**0.9**–**3.0)**
Never use	**67.3** (**65.8**–**68.8)**	**22.8** (**21.7**–**23.9)**	**7.6** (**6.9**–**8.5)**	**1.4** (**1.2**–**1.8)**	**0.8** (**0.6**–**1.2)**
Tobacco products /nicotine Vaping					
≤17^b^	7.4 (6.9–7.9)	24.9 (24.1–25.7)	27.7 (27.1–28.5)	20.5 (19.9–21.3)	19.5 (18.8–20.2)
18–20	8.3 (7.3–9.5)	**31.4** (**29.9**–**33.0)**	29.0 (27.6–30.5)	19.2 (18.0–20.4)	**12.1** (**11.2**–**13.0)**
21–29	7.4 (6.0–9.1)	**34.5** (**32.6**–**36.5)**	**30.9** (**28.5**–**33.5)**	**16.8** (**15.3**–**18.6)**	**10.3** (**9.1**–**11.7)**
≥30	10.6 (7.1–15.5)	29.1 (24.8–33.7)	31.7 (27.1-36.6)	17.5 (14.7–20.7)	**11.2** (**8.4**–**14.7)**
Never use	**36.0** (**35.1**–**37.0)**	**41.1** (**40.1**–**42.0)**	**16.7** (**16.0**–**17.5)**	**4.9** (**4.5**–**5.2)**	**1.3** (**1.2**–**1.6)**
Cannabis					
≤17^b^	3.9 (3.5–4.4)	18.0 (17.0–19.0)	25.8 (24.8–26.8)	24.9 (23.9–26.0)	27.4 (26.4–28.4)
18–20	**5.1** (**4.3**–**6.1)**	**26.5** (**25.3**–**27.9)**	**29.8** (**28.3**–**31.4)**	**22.5** (**21.1**–**23.9)**	**16.1** (**15.0**–**17.2)**
21–29	**5.8** (**4.9**–**7.0)**	**29.1** (**17.2**–**31.0)**	**31.7** (**29.6**–**33.9)**	**21.6** (**20.0**–**23.2)**	**11.8** (**10.6**–**13.1)**
≥30	5.0 (3.3–7.7)	**26.4** (**23.2**–**29.9)**	**36.8** (**33.2**–**40.6)**	**19.7** (**16.9**–**22.9)**	**12.0** (**9.8**–**14.8)**
Never use	**31.0** (**30.1**–**31.9)**	**42.2** (**41.2**–**43.1)**	**20.1** (**19.3**–**20.9)**	**5.4** (**5.1**–**5.9)**	**1.4** (**1.2**–**1.5)**
Hallucinogens					
≤17^b^	3.2 (2.5–4.0)	13.6 (11.5–15.9)	21.4 (19.4–23.6)	22.7 (20.6–25.0)	39.1 (36.6–41.6)
18–20	2.9 (2.2–3.9)	14.8 (13.1–16.6)	22.7 (20.4–25.1)	25.0 (23.0–27.0)	**34.7** (**32.5**–**37.0)**
21–29	3.8 (2.7–5.4)	16.0 (13.7–18.6)	23.3 (20.8–26.1)	24.4 (22.3–26.6)	**32.5** (**30.4**–**34.6)**
≥30	2.7 (1.6–4.4)	11.6 (8.9–14.9)	21.0 (16.3–26.6)	26.0 (20.3–32.7)	38.8 (34.3–43.4)
Never use	**21.0** (**20.4**–**21.7)**	**36.4** (**35.8**–**37.0)**	**24.8** (**24.2**–**25.4)**	**12.2** (**11.8**–**12.7)**	**5.5** (**5.3**–**5.7)**
Cocaine					
≤17^b^	2.7 (1.8–4.0)	12.6 (10.2–15.6)	23.0 (20.5–25.8)	22.5 (20.0–25.3)	39.1 (35.8–42.6)
18–20	2.7 (2.0–3.6)	**16.4** (**14.4**–**18.6)**	22.0 (19.5–24.8)	22.0 (19.7–24.5)	36.9 (33.9–40.0)
21–29	3.7 (2.8–5.1)	**16.7** (**14.9**–**18.7)**	24.3 (22.0–26.8)	22.0 (19.9–24.3)	**33.2** (**31.1**–**35.5)**
≥30	4.7 (2.9–7.6)	**17.2** (**14.2**–**20.7)**	**28.3** (**24.2**–**32.8)**	23.3 (19.5–27.5)	**26.5** (**23.4**–**30.0)**
Never use	**20.5** (**19.9**–**21.2)**	**35.6** (**35.0**–**36.1)**	24.4 (23.9–25.0)	**12.9** (**12.5**–**13.4)**	**6.5** (**6.3**–**6.8)**
Methamphetamine					
≤17^b^	2.7 (1.8–4.1)	13.1 (9.2–18.4)	23.0 (19.5–27.0)	20.9 (17.3–25.1)	40.3 (36.1–44.6)
18–20	3.8 (2.4–6.0)	17.3 (14.0–21.1)	23.5 (19.3–28.4)	21.6 (17.9–26.0)	**33.7** (**29.7**–**38.1)**
21–29	**4.7** (**3.1**–**7.2)**	12.6 (10.1–15.5)	23.1 (19.4–27.3)	18.9 (16.1–22.2)	40.7 (35.4–46.2)
≥30	1.6 (0.7–4.0)	11.0 (8.0–15.0)	20.1 (15.5–25.7)	21.1 (15.8–27.5)	46.2 (40.5–51.9)
Never use	**18.7** (**18.1**–**19.3)**	**33.6** (**33.1**–**34.1)**	24.4 (23.9–25.0)	**14.1** (**13.6**–**14.5)**	**9.2** (**8.9**–**9.5)**
Heroin					
≤17^b^	2.2 (0.9–5.3)	9.0 (5.6–14.1)	26.5 (17.8–37.5)	19.6 (13.4–27.9)	42.7 (32.3–53.7)
18–20	2.6 (1.3–5.5)	14.7 (9.2–22.5)	17.9 (12.1–25.7)	18.5 (12.4–26.8)	46.3 (38.0–54.8)
21–29	1.9 (1.1–3.4)	13.1 (8.2–20.5)	19.8 (16.4–23.8)	20.8 (16.6–25.9)	44.3 (38.4–50.4)
≥30	1.9 (0.7–5.1)	6.3 (3.7–10.6)	22.3 (16.9–28.8)	18.9 (14.2–24.8)	50.7 (43.3–58.0)
Never use	**18.1** (**17.6**–**18.7)**	**32.9** (**32.4**–**33.4)**	24.4 (23.9–25.0)	14.3 (13.9–14.8)	**10.2** (**9.9**–**10.6)**
Inhalants					
≤17 ^b^	6.2 (5.2–7.5)	18.4 (16.3–20.8)	23.6 (21.3–26.0)	20.4 (18.5–22.5)	31.3 (28.9–33.9)
18–20	**3.7** (**2.5**–**5.2)**	15.8 (12.4–19.9)	24.3 (20.9–28.0)	21.4 (18.8–24.3)	34.9 (31.8–38.2)
21–29	**2.0** (**1.1**–**3.6)**	**14.2** (**11.5**–**17.6)**	22.5 (18.9–26.5)	20.1 (17.2–23.3)	**41.2** (**36.1**–**46.5)**
≥30	3.5 (1.7–6.9)	17.0 (11.7–24.1)	25.1 (17.5–34.7)	**11.9** (**7.9**–**17.6)**	**42.5** (**33.3**–**52.2)**
Never use	**19.1** (**18.5**–**19.7)**	**34.0** (**33.5**–**34.5)**	24.4 (23.9–24.9)	**13.9** (**13.4**–**14.3)**	**8.6** (**8.3**–**8.9)**

Data source: 2022–2023 National Surveys on Drug Use and Health.

^a^Different substances include alcohol, tobacco products or nicotine vaping, cannabis, opioids (prescription (Rx) opioids, heroin, or illegally made fentanyl), Rx stimulants, Rx sedatives or tranquilizers, hallucinogens, cocaine, methamphetamine, and inhalants. Bold prevalence was statistically significantly different from the corresponding estimate of the reference group within each table cell (*P* < 0.05). ^b^Reference group.

^c^Age of first misuse was unavailable for non-past-year initiates of Rx medication misuse.

### Polysubstance use disorders by age of initiation of substance use and never use

Among adults who initiated using any substance before age 18, 9.7% had ≥2 SUDs in the past year (Table 3), while among those who initiated at age ≥30, only 0.9% had ≥2 SUDs. Specifically, past-year prevalence of ≥2 SUDs was 10.7% among adults who initiated alcohol use before age 18, compared with 1.3% among those who never used alcohol; it was 11.5% among those who initiated tobacco products or vaping nicotine before age 18, compared with 0.6% among who never used them; and it was 16.8% among those who initiated cannabis use before age 18, compared with 1.1% among those who never used it. These results are highly consistent with those after age- and sex-adjustment (results available upon request).

**Table 3:** Prevalence of the number of past-year substance use disorders (SUDs)^a^ among adults in the US overall and by age of initiation of overall and specific substance use, weighted percentage (95% CI) (N = 92,233).

Factors	0 SUD	1 SUD	2 SUDs	≥3 SUDs
Overall	76.2 (75.7–76.6)	17.6 (17.2–18.0)	4.6 (4.4–4.9)	1.6 (1.5–1.8)

Age of First Use of Any Substance^c^				
Age of first use of any substance				
≤17 ^b^	66.3 (65.6–67.0)	24.0 (23.4–24.6)	7.1 (6.7–7.6)	2.6 (2.4–2.9)
18–20	**83.2** (**82.1**–**84.3)**	**14.3** (**13.4**–**15.3)**	**2.1** (**1.8**–**2.3)**	**0.4** (**0.3**–**0.7)**
21–29	**90.9** (**89.8**–**91.9)**	**7.7** (**6.8**–**8.6)**	**1.2** (**0.9**–**1.7)**	**0.2** (**0.1**–**0.4)**
≥30	**95.5** (**93.7**–**96.8)**	**3.6** (**2.4**–**5.4)**	**0.6** (**0.3**–**1.3)**	**0.3** (**0.1**–**1.4)**

Age of First Substance Use by Substance^c^				
Alcohol				
≤17 ^b^	64.8 (64.0–65.6)	24.5 (23.8–25.2)	7.8 (7.3–8.3)	2.9 (2.6–3.2)
18–20	**80.0** (**79.0**–**81.1)**	**16.3** (**15.3**–**17.3)**	**2.9** (**2.6**–**3.2)**	**0.8** (**0.6**–**1.1)**
21–29	**85.6** (**84.4**–**86.6)**	**11.8** (**10.9**–**12.7)**	**2.1** (**1.8**–**2.6)**	**0.5** (**0.4**–**0.8)**
≥30	**90.5** (**87.7**–**92.7)**	**7.6** (**5.6**–**10.2)**	**1.7** (**0.8**–**3.7)**	**0.2** (**0.0**–**1.3)**
Never use	**91.8** (**90.8**–**92.8)**	**6.9** (**6.0**–**7.9)**	**1.0** (**0.8**–**1.3)**	**0.3** (**0.1**–**0.5)**
Tobacco products/nicotine vaping				
≤17 ^b^	62.4 (61.5–63.3)	26.0 (25.2–26.9)	8.3 (7.8–9.0)	3.2 (2.9–3.6)
18–20	**72.0** (**70.0**–**73.3)**	**22.2** (**21.0**–**23.4)**	**4.6** (**4.2**–**5.1)**	**1.2** (**1.0**–**1.5)**
21–29	**75.0** (**72.9**–**77.1)**	**19.9** (**18.1**–**21.7)**	**4.1** (**3.5**–**4.9)**	**1.0** (**0.6**–**1.5)**
≥30	**83.8** (**80.5**–**86.7)**	**12.4** (**9.7**–**15.7)**	**2.5** (**1.6**–**3.9)**	**1.3** (**0.5**–**3.0)**
Never use	**93.6** (**93.0**–**94.1)**	**5.8** (**5.3**–**6.3)**	**0.6** (**0.5**–**0.8)**	**0.0** (**0.0**–**0.1)**
Cannabis				
≤17 ^b^	52.9 (51.7–54.1)	30.3 (29.3–31.4)	11.9 (11.1–12.8)	4.9 (4.4–5.4)
18–20	**68.9** (**67.4**–**70.4)**	**24.2** (**22.9**–**25.7)**	**5.6** (**5.0**–**6.3)**	**1.3** (**1.0**–**1.6)**
21–29	**74.8** (**72.8**–**76.7)**	**19.8** (**18.3**–**21.5)**	**4.2** (**3.5**–**5.2)**	**1.1** (**0.7**–**1.8)**
≥30	**79.1** (**76.8**–**81.2)**	**17.7** (**15.6**–**20.0)**	**2.4** (**1.8**–**3.3)**	**0.8** (**0.4**–**1.5)**
Never use	**90.0** (**89.6**–**90.5)**	**8.9** (**8.5**–**9.4)**	**0.9** (**0.8**–**1.0)**	**0.2** (**0.1**–**0.3)**
Hallucinogens				
≤17 ^b^	42.9 (41.1–44.9)	32.0 (29.9–34.2)	16.0 (14.4–17.8)	9.1 (7.7–10.7)
18–20	**49.2** (**46.6**–**51.8)**	31.0 (29.0–33.2)	14.2 (12.6–16.0)	**5.6** (**4.6**–**6.9)**
21–29	**53.3** (**51.4**–**55.1)**	30.3 (28.4–32.2)	**11.7** (**10.0**–**13.6)**	**4.8** (**3.9**–**5.9)**
≥30	47.9 (41.9–53.9)	35.6 (30.8–40.7)	13.0 (9.2–18.0)	**3.5** (**2.3**–**5.5)**
Never use	**82.6** (**82.2**–**83.0)**	**14.4** (**14.0**–**14.8)**	**2.5** (**2.3**–**2.7)**	**0.5** (**0.4**–**0.6)**
Cocaine				
≤17 ^b^	38.9 (35.1–42.8)	32.5 (30.0–35.7)	16.0 (14.1–18.2)	12.5 (10.6–14.7)
18–20	**45.5** (**42.2**–**48.8)**	31.8 (28.8–34.9)	15.7 (13.4–18.2)	**7.1** (**5.8**–**8.6)**
21–29	**52.9** (**50.4**–**55.5)**	**28.8 (26.9**–**30.7)**	**12.8 (11.3**–**14.6)**	**5.4 (4.5**–**6.6)**
≥30	**54.0 (50.3**–**57.7)**	31.2 **(**26.9–36.0)	**11.0 (8.1**–**14.7)**	**3.8 (2.6**–**5.6)**
Never use	**81.6 (81.2**–**82.1)**	**15.0 (14.6**–**15.5)**	**2.8 (2.6**–**3.0)**	**0.5 (0.4**–**0.6)**
Methamphetamine				
≤17 ^b^	36.2 **(**31.5–41.2)	31.8 **(**26.9–37.2)	18.8 **(**16.1–21.9)	13.2 **(**10.7–16.2)
18–20	**48.4 (44.4**–**52.5)**	28.9 **(**24.6–33.5)	**12.3 (10.2**–**14.7)**	10.4 **(**8.0–13.5)
21–29	42.7 **(**37.5–48.1)	28.2 **(**25.0–31.6)	17.6 **(**14.2–21.7)	11.5 **(**8.9–14.7)
≥30	32.8 **(**27.2–39.0)	33.6 **(**28.0–39.6)	22.4 **(**18.0–27.4)	11.3 **(**8.6–14.7)
Never use	**78.6 (78.1**–**79.0)**	**16.7 (16.4**–**17.2)**	**3.8 (3.6**–**4.0)**	**0.9 (0.8**–**1.1)**
Heroin				
≤17 ^b^	39.8 **(**29.7–50.8)	23.0 **(**16.3–31.5)	14.0 **(**9.4–20.3)	23.2 **(**15.7–32.8)
18–20	35.5 **(**27.0–45.1)	24.7 **(**18.9–31.7)	**23.1 (17.4**–**30.0)**	16.7 **(**12.1–22.5)
21–29	36.2 **(**29.5–43.4)	29.2 **(**24.2–34.7)	16.2 **(**12.6–20.5)	18.4 **(**15.0–22.5)
≥30	**22.8 (16.3**–**31.0)**	**34.3 (28.8**–**40.2)**	**23.1 (17.5**–**29.8)**	19.8 **(**15.2–25.4)
Never use	**77.3 (76.9**–**77.8)**	17.3 **(**16.9–17.7)	**4.3 (4.0**–**4.5)**	**1.1 (1.0**–**1.3)**
Inhalants				
≤17 ^b^	49.6 **(**47.4–51.8)	29.4 **(**27.0–31.9)	13.4 **(**11.8–15.1)	7.7 **(**6.3–9.3)
18–20	50.5 **(**46.4–54.5)	29.0 **(**25.6–32.7)	12.6 **(**10.2–15.5)	7.9 **(**6.2–10.1)
21–29	45.4 **(**40.5–50.4)	**34.3 (30.9**–**37.9)**	13.5 **(**10.7–17.0)	6.7 **(**5.3–8.5)
≥30	51.2 **(**42.3–59.9)	29.3 **(**22.5–37.1)	14.8 **(**9.7–22.0)	4.7 **(**2.7–8.0)
Never use	**79.1 (78.6**–**79.5)**	**16.2 (15.8**–**16.7)**	**3.7 (3.5**–**4.0)**	**1.0 (0.9**–**1.1)**

Data source: 2022–2023 National Surveys on Drug Use and Health (NSDUH).

^a^Substance use disorders include past-year alcohol use disorder, cannabis use disorder, opioid use disorder, prescription (Rx) stimulant use disorder, Rx sedative or tranquilizer use disorder, cocaine use disorder, hallucinogen use disorder, methamphetamine use disorder, inhalant use disorder, or past-month nicotine dependence. Bold prevalence was statistically significantly different from the corresponding estimate of the reference group within each table cell (*P* < 0.05).

^b^Reference group.

^c^Age of first misuse was unavailable for non-past-year initiates of Rx medication misuse.

### Multivariable results on the number of different substances used/misused

After controlling for covariates (Table 4), the number of different substances used or misused in the past year was 23–42% lower among adults who initiated substance use in adulthood than among those who initiated earlier (incidence density ratio (IDR) range=0.58–0.77, 95% CIs=0.53–0.79). Specifically (Table 5), compared with corresponding adult counterparts who initiated before age 18, the number of different substances used was 5% lower (IDR = 0.95, 95% CI = 0.93–0.97) among those who initiated alcohol at ages 21–29 and 64% lower (IDR = 0.36, 95% CI = 0.34–0.38) among those who never used alcohol; 4% lower (IDR = 0.96, 95% CIs=0.93–0.99) among those who initiated cannabis at ages 18–29, 28% lower (IDR = 0.72, 95% CI = 0.70–0.74) among those who never used it, but 8% higher (IDR = 1.08, 95% CI = 1.03–1.13) among those who initiated at age ≥30. Compared with counterparts with age of initiation before 18, the number of different substances used was similar among adults who initiated tobacco/nicotine vaping, hallucinogen, or methamphetamine use at ages 18–29, 10–13% higher (IDR range=1.10–1.13, 95% CIs=1.03–1.20) among adults who initiated it at age ≥30, but 6–28% lower (IDR range=0.72–0.94, 95% CIs=0.71–0.98) among those who never used the corresponding substance.

**Table 4:** After adjusting for covariates, results of 3 multivariable Poisson regression models show age of any substance use initiation associated with: (1) the number of different substances used or misused in the past year (secondary outcome); (2) the number of substance use disorders (SUDs, primary outcome); (3) the number of moderate-severe SUDs (secondary outcome) among adults in the US.

Age of any substance use initiation	Model 1 outcome: Number of different substances used/misused Incidence Density Ratio (95% CI) (N = 92,233)	Model 2 outcome: Number of SUDs Incidence Density Ratio (95% CI) (N = 92,233)	Model 3 outcome: Number of moderate-severe alcohol or drug use disorders Incidence Density Ratio (95% CI) (N = 92,233)
Age of first use of any substance^b^			
≤17 ^a^	1.00	1.00	1.00
18–20	**0.77 (0.76**–**0.79)**	**0.50 (0.47**–**0.54)**	**0.41 (0.36**–**0.46)**
21–29	**0.61 (0.59**–**0.62)**	**0.27 (0.24**–**0.31)**	**0.16 (0.13**–**0.21)**
≥30	**0.58 (0.53**–**0.62)**	**0.17 (0.12**–**0.24)**	**0.18 (0.09**–**0.35)**

Data source: 2022–2023 National Surveys on Drug Use and Health.

^a^Reference group.

^b^Different substances include alcohol, tobacco products or nicotine vaping, cannabis, opioids (prescription (Rx) opioids, heroin, or illegally made fentanyl), Rx stimulants, Rx sedatives or tranquilizers, hallucinogens, cocaine, methamphetamine, or inhalants. Substance use disorders include past-year alcohol use disorder, cannabis use disorder, opioid use disorder, Rx stimulant use disorder, Rx sedative or tranquilizer use disorder, cocaine use disorder, hallucinogen use disorder, methamphetamine use disorder, inhalant use disorder, or past-month nicotine dependence. Bold prevalence was statistically significantly different from the estimate of the reference group within each table cell (*P* < 0.05).

**Table 5:** After adjusting for covariates,^c^ results of 3 multivariable Poisson regression models show age of specific substance use initiation associated with: (1) the number of different substances used or misused in the past year (secondary outcome); (2) the number of substance use disorders (SUDs) (primary outcome); (3) the number of moderate-severe SUDs (secondary outcome) among adults in the US.

Age of specific substance use initiation	Model 4 outcome: Number of different substances used/misused Incidence Density Ratio (95% CI) (N = 92,233)	Model 5 outcome: Number of SUDs Incidence Density Ratio (95% CI) (N = 92,233)	Model 6 outcome: Number of moderate-severe alcohol or drug use disorders Incidence Density Ratio (95% CI) (N = 92,233)
Age of First Substance Use by Substance^b^			
Alcohol			
≤17 ^a^	1.00	1.00	1.00
18-20	1.00 (0.99–1.02)	**0.93 (0.89**–**0.97)**	**0.89 (0.74**–**0.99)**
21–29	**0.95 (0.93**–**0.97)**	**0.84 (0.77**–**0.92)**	**0.68 (0.57**–**0.83)**
≥30	0.93 (0.86–1.01)	0.91 (0.69–1.19)	0.79 (0.41–1.53)
Never use	**0.36 (0.34**–**0.38)**	**0.71 (0.62**–**0.81)**	**0.55 (0.40**–**0.76)**
Tobacco products/nicotine vaping			
≤17 ^a^	1.00	1.00	1.00
18–20	0.98 (0.96–1.00)	0.96 (0.90–1.02)	1.01 (0.93–1.11)
21–29	0.99 (0.97–1.02)	0.95 (0.87–1.03)	1.02 (0.89–1.17)
≥30	**1.10 (1.03**–**1.18)**	0.80 (0.62–1.04)	1.05 (0.76–1.46)
Never use	**0.72 (0.71**–**0.73)**	**0.35 (0.31**–**0.38)**	**0.45 (0.36**–**0.55)**
Cannabis			
≤17 ^a^	1.00	1.00	1.00
18–20	**0.96 (0.94**–**0.98)**	**0.88 (0.83**–**0.93)**	**0.84 (0.76**–**0.93)**
21–29	**0.96 (0.93**–**0.99)**	**0.86 (0.79**–**0.93)**	**0.79 (0.69**–**0.90)**
≥30	**1.08 (1.03**–**1.13)**	**0.85 (0.76**–**0.95)**	0.91 (0.70–1.18)
Never use	**0.72 (0.70**–**0.74)**	**0.54 (0.50**–**0.58)**	**0.38 (0.31**–**0.45)**
Hallucinogens			
≤17 ^a^	1.00	1.00	1.00
18–20	0.99 (0.95–1.03)	0.98 (0.91–1.06)	0.95 (0.86–1.05)
21–29	0.99 (0.96–1.02)	1.01 (0.94–1.09)	1.06 (0.92–1.25)
≥30	**1.13 (1.07**–**1.20)**	1.13 (0.96–1.32)	1.18 (0.88–1.59)
Never use	**0.83 (0.80**–**0.85)**	**0.74 (0.69**–**0.79)**	**0.65 (0.57**–**0.75)**
Cocaine			
≤17 ^a^	1.00	1.00	1.00
18–20	0.99 (0.95–1.03)	1.04 (0.94–1.16)	1.12 (0.95–1.32)
21–29	0.99 (0.94–1.04)	0.99 (0.90–1.09)	1.03 (0.86–1.24)
≥30	0.95 (0.90–1.01)	1.01 (0.87–1.16)	0.95 (0.70–1.29)
Never use	**0.87 (0.84**–**0.91)**	**0.76 (0.69**–**0.84)**	**0.64 (0.54**–**0.75)**
Methamphetamine			
≤17 ^a^	1.00	1.00	1.00
18–20	0.96 (0.90–1.02)	**0.89 (0.80**–**0.99)**	0.92 (0.72–1.19)
21–29	1.01 (0.94–1.08)	0.97 (0.84–1.11)	0.95 (0.76–1.18)
≥30	**1.12 (1.05**–**1.19)**	1.07 (0.93–1.23)	0.97 (0.73–1.28)
Never use	**0.94 (0.90**–**0.98)**	**0.83 (0.76**–**0.91)**	**0.77 (0.64**–**0.93)**
Heroin			
≤17 ^a^		1.00	1.00
18–20		0.95 (0.75–1.20)	0.81 (0.60–1.09)
21–29		0.96 (0.74–1.25)	0.87 (0.65–1.15)
≥30		1.11 (0.83–1.49)	1.24 (0.83–1.86)
Never use		0.82 (0.65–1.03)	**0.68 (0.52**–**0.89)**
Inhalants			
≤17 ^a^	1.00	1.00	1.00
18–20	**1.05 (1.003-1.10)**	1.00 (0.90–1.11)	1.06 (0.88–1.28)
21–29	**1.10 (1.05**–**1.15)**	1.08 (0.96–1.23)	0.99 (0.82–1.20)
≥30	**1.20 (1.09**–**1.33)**	1.14 (0.90–1.44)	1.23 (0.88–1.72)
Never use	**0.95 (0.92**–**0.97)**	**0.88 (0.83**–**0.93)**	**0.75 (0.67**–**0.85)**

Data source: 2022–2023 National Surveys on Drug Use and Health.

^a^Reference group.

^b^Age of first misuse was unavailable for non-past-year initiates of prescription medication misuse. Different substances include alcohol, tobacco products or nicotine vaping, cannabis, opioids (prescription (Rx) opioids, heroin, or illegally made fentanyl), Rx stimulants, Rx sedatives or tranquilizers, hallucinogens, cocaine, methamphetamine, or inhalants. Substance use disorders include past-year alcohol use disorder, cannabis use disorder, opioid use disorder, Rx stimulant use disorder, Rx sedative or tranquilizer use disorder, cocaine use disorder, hallucinogen use disorder, methamphetamine use disorder, inhalant use disorder, or past-month nicotine dependence. Bold prevalence was statistically significantly different from the estimate of the reference group within each table cell (*P* < 0.05).

^c^Associations between all covariates and the 3 outcomes can be found in Supplementary Table 3.

### Multivariable results on the number of SUDs

After adjusting for covariates (Table 4), the number of SUDs was 50–83% lower among adults who initiated substance use during adulthood than before age 18 (IDR range=0.17–0.50, 95% CIs=0.12–0.54). Specifically (Table 5), compared with corresponding adult counterparts who initiated before age 18, the number of SUDs was 7–12% lower (IDR range=0.88–0.93, 95% CIs=0.83–0.97) among those who initiated alcohol or cannabis at ages 18–20 and 14–16% lower (IDR range=0.84–0.86, 95% CIs=0.77–0.93) among those who initiated alcohol or cannabis at ages 21–29; it was 12–26% lower (IDR range=0.74–0.88, 95% CIs=0.69–0.93) among those who never initiated hallucinogens, cocaine, methamphetamine, or inhalants, and 29–65% lower (IDR range=0.31–0.71, 95% CIs=0.31–0.81) among those who never initiated alcohol, cannabis, or tobacco/nicotine use.

### Multivariable results on the number of moderate-severe SUDs

Multivariable results (Table 4) showed that the number of moderate-severe SUDs was 59–82% lower among adults who initiated substance use during adulthood than before age 18 (IDR range=0.18–0.41, 95% CIs=0.09–0.46). Specifically, compared with corresponding adult counterparts who initiated before age 18 (Table 5), the number of moderate-severe SUDs was 32% lower for those initiating alcohol at ages 21–29 (IDR = 0.68, 95% CI = 0.57–0.83), 21% lower among those initiating cannabis at ages 21–29 (IDR = 0.79, 95% CI = 0.69–0.90), 23–36% lower (IDR range=0.64–0.77, 95% CIs=0.54–0.93) among those who never initiated hallucinogen, heroin, cocaine, inhalants, or methamphetamine, and 45–62% lower (IDR range=0.38–0.55, 95% CIs=0.31–0.76) among those who never initiated alcohol, cannabis, or tobacco/nicotine use.

Multivariable results also show that the number of moderate-severe SUDs (Supplementary Table 3) was associated with being aged 18–29, male, non-Hispanic American Indian or Alaska Native (AIAN) or Black race/ethnicity, having <high school or some college education, an annual family income <$20,000, being unemployed or divorced/separated/never married, residing in large metropolitan areas, self-reporting less than excellent health, and having emergency room visit(s), suicidal ideation, and MDE. Similar results were found for overall polysubstance use disorders.

## Discussion

We found that use of multiple substances as well as overall and moderate-severe polysubstance use disorders were common among US adults with substance use. Specifically, we found that over half of adults who misused opioids in the past year used ≥4 substances; among adults with opioid use disorder, nearly one-third had ≥3 SUDs. Among adults who used hallucinogens, cocaine, or methamphetamine in the past year, the vast majority used ≥4 substances, and among adults with past-year hallucinogen, cocaine, or methamphetamine use disorder, 48.2–72.0% had ≥3 SUDs. Consistently, nearly half of US drug overdose deaths involved multiple substances in 2022 [5]. Despite a decrease in overall overdose deaths in 2023, polysubstance overdose mortality has continued to increase (e.g., higher number and rate of overdose deaths involving opioids plus cocaine or methamphetamine) [5, 23], further exacerbating the complexity of the overdose crisis (involving more than one substance) and highlighting the urgency of addressing polysubstance use and use disorders.

The observed patterns of polysubstance use disorders may indicate elevated biological and environmental vulnerabilities [2, 8–15, 35–39] among individuals with these conditions, reflecting the critical need for comprehensive screening and intervention. Furthermore, findings on associations between age of substance use initiation and overall and moderate-severe polysubstance use disorders, underscore the importance of implementing evidence-based primary prevention strategies targeting youth throughout adulthood.

Notably, alcohol, cannabis, and nicotine are commonly used substances, contributing to morbidity, mortality, and long-term social-behavioral consequences [18, 45–50], which are likely exacerbated by polysubstance use disorders. Although causal relationships cannot be established, our results underscore the critical need for evidence-based targeted prevention strategies for avoiding alcohol, nicotine, and cannabis consumption—particularly before age 21 —and minimizing their consumption thereafter. These results are highly consistent with the established theories that early substance use interacts with neurobiological vulnerabilities and developmental immaturity in reward, control, and stress‑regulation systems, increasing sensitivity to reinforcement and weakening cognitive and emotional regulation [28–30, 35–37]. Combined with environmental reinforcement and strengthened associative learning [38, 39], these factors create a developmental cascade that elevates the risk of progressing from single‑ to polysubstance use. Taken together, our detailed epidemiological findings help inform data-driven, specific prevention messages. Such prevention efforts may help substantially lower the risk for polysubstance use disorders among adults in the US.

Alcohol is the most prevalent substance used in the US, with a minimum legal drinking age of 21 [51]. Our results of multivariable regression analyses consistently show strong associations between the age of alcohol use initiation and the number of different substances used and polysubstance use disorders. Compared with initiation before age 18, after adjusting for potential confounding factors, alcohol initiation at ages 21–29 was consistently associated with fewer substances used and fewer overall and moderate-severe SUDs. Compared with adults who initiated alcohol before age 18, those who never used alcohol had 64% fewer different substances used, 29% fewer SUDs, and 45% fewer moderate-severe SUDs. These findings suggest that delaying or preventing the initiation of alcohol use—through targeted modifications of risk factors beginning in adolescence and continuing into adulthood—may offer critical leverage points for preventing early onset as well as overall and moderate-severe polysubstance use disorders.

The minimum age for nonmedical cannabis use is 21 in legal cannabis states, where most Americans reside [52]. Compared with initiation before age 18, our study shows that initiation of cannabis use at ages 21–29 was consistently associated with reduced risk for the number of different substances used, polysubstance use disorders, and moderate-severe polysubstance use disorders. Moreover, among adults who never used cannabis, the number of SUDs was 46% lower, and the number of moderate-severe SUDs was 62% lower, compared with their counterparts who initiated use before age 18. Consistent with the alcohol-related results discussed above, findings suggest that delaying or preventing the initiation of cannabis use—via targeted risk-factor prevention and interventions from adolescence through adulthood—may provide critical leverage for preventing early onset as well as overall and moderate-severe polysubstance use disorders.

Similarly, our results suggest associations between tobacco/nicotine use initiation and polysubstance use disorders (overall and moderate-severe). Under US Federal law, 21 is the legal age for tobacco use to reduce youth access [53]. Our results suggest that among adults who never used tobacco/nicotine, the number of different substances used in the past year was 28% lower, the number of SUDs was 65% lower, and the number of moderate-severe alcohol or drug use disorders was 55% lower, compared with their counterparts who initiated use before age 18. Yet, initiation of tobacco or nicotine use at any age appears problematic in relation to both overall and moderate-severe polysubstance use disorders. This may be attributable to nicotine’s highly addictive nature [54], its overlap with other substance use and reward-potentiating effects on other substances [3, 21–30], and the associations between nicotine dependence and persistent co‑occurring SUDs [21, 24–27].

Our findings on moderate-severe polysubstance use disorders also suggest that substance use prevention efforts benefit from including adults as well. Specifically, compared to initiation before age 18, initiation of hallucinogen, cocaine, heroin, methamphetamine, or inhalant use at age ≥18 or initiation of alcohol or cannabis use at ≥30 was not associated with fewer moderate-severe SUDs. Thus, our results suggest that targeted risk‑factor prevention and interventions initiated in adolescence and maintained through adulthood may be critical. Clinically, patients who initiated using these drugs during adulthood could still benefit from prevention efforts for moderate-severe polysubstance use disorders. Future research is needed to examine whether and how the risk factors for moderate-severe polysubstance use disorders linked to early initiation (before age 18) differ from those associated with late initiation (age ≥30).

Our findings on the prevalence of specific combinations of and correlates of polysubstance use disorders help inform screening and treatment efforts and tailored patient-centered clinical interventions. Clinical screening efforts for SUDs often focus on patients with early substance use initiation [54]. As anticipated [55], the most commonly used substances—alcohol, cannabis, and nicotine—and corresponding SUDs frequently co-occur with other drug use disorders. Treatment approaches that account for these SUDs may have multiple benefits, as shown in recent findings indicating that tobacco cessation may enhance recovery outcomes from other co-occurring SUDs [56]. Validated, electronic brief screening and assessment tools are available and can be embedded in electronic health records to help detect polysubstance use and related use disorders [57]. Consistent with high overdose mortality rates among non-Hispanic AIAN and Black adults [58–61], our multivariable results also show that the number of moderate-severe SUDs was markedly higher in these 2 subpopulations than in non-Hispanic White adults. These results suggest the value of incorporating strategies that directly address polysubstance use disorders in substance use interventions and policies for these populations.

Although high prevalence of polysubstance use disorders may contribute to poor treatment outcomes and high mortality [1–7, 62, 63], their treatment is uniquely challenging. It is necessary to emphasize low-barrier access to patient-centered care through working with patients to set their own treatment goals and prioritizing engagement, stabilization, and functional recovery. Moreover, our results suggest the importance of tailored medical and behavioral interventions for shared reward pathways across substances [2]. Patients may still regard it as acceptable to start treatment for one of their SUDs, even if they are unwilling to address the full range of SUDs simultaneously. Although comprehensive interventions focusing on multiple disorders are often warranted, treatment of even one SUD may confer meaningful benefits in reducing polysubstance use disorders [26, 27]. Remission of one SUD may increase the likelihood of remission of other SUDs and decrease the probability of subsequent onset of new SUDs [26, 27]. Furthermore, research is needed to develop effective treatment mechanisms for polysubstance use disorders (e.g., potential therapeutic benefits of glucagon-like peptide 1 (GLP-1) medications) [64, 65]. Future studies are also warranted to delineate the patterns of polysubstance use disorders and their co-occurrence with other mental disorders, thereby informing the development of more integrated treatment approaches. Finally, effectively addressing polysubstance use disorder requires coordinated care across providers (e.g., through integrated care models), peer navigation, and attention to co-occurring mental health conditions [66] and other risk factors [19, 67].

### Limitations

This study has several limitations. First, the cross-sectional nature of NSDUH data precludes drawing causal relationships. The use of specific substances may be bidirectionally related to the use of other substances or influenced by additional factors. Also, NSDUH does not collect lifetime SUDs. Future research is needed to fully understand why certain combinations of SUDs co-occur more or less frequently. For example, findings may be strengthened by examining data from the Adolescent Brain Cognitive Development (ABCD) Study, the largest longitudinal investigation of brain and cognitive development in US youth [68]. Second, NSDUH is self-reported and subject to recall and social-desirability bias, and it had low survey response rates during the COVID pandemic. Third, we may underestimate the prevalence of polysubstance use disorders because NSDUH excluded unhoused adults not living in shelters and institutionalized populations who may have a higher prevalence than the general population [69, 70], because NSDUH did not collect past-year tobacco use disorder based on the DSM-5 diagnostic criteria among adults with tobacco or nicotine use, and because use of different substances could occur unintentionally [71]. Fourth, the age of substance use initiation can be impacted by individual characteristics (e.g., genetics, impulsivity, sensation-seeking, anxiety), social-environmental influences (e.g., peer pressure, culture, substance availability), and family history/dynamics [72, 73] that cannot be examined with NSDUH data.

## Conclusions

Overall and moderate-severe polysubstance use disorders are common among US adults with substance use, aligning with the recent rise in polysubstance overdose mortality. Attention to polysubstance use disorders and their specific combinations is valuable when planning and implementing SUD treatment—emphasizing low-barrier access, integrated care models, and tailored, patient-centered approaches to address individuals’ complex needs, preferences, and treatment goals. Associations of polysubstance use disorders with age initiations of specific substances underscore the importance of prevention efforts for avoiding alcohol, nicotine, and cannabis consumption, particularly among those aged <21, and minimizing their use thereafter.

## Supplementary information

Supplemental/Online Materials

## Data Availability

This study was based on public-use files. Both data and the data dictionary are accessible to the public at: https://www.datafiles.samhsa.gov/dataset/national-survey-drug-use-and-health-2022-nsduh-2022-ds0001.

## Access to data and data analysis

Dr. Beth Han had full access to all the data in the study and takes responsibility for the integrity of the data and the accuracy of the data analysis.
