# Supplementary material for: Polysubstance use disorders among US adults
Source: Mol Psychiatry. 2026 Apr 29;31(9):5303–15. doi: 10.1038/s41380-026-03618-z (PMC13442028; doi:10.1038/s41380-026-03618-z)
Supplement: Supplementary file 1 [file 41380_2026_3618_MOESM1_ESM.pdf]

Supplemental/Online Materials

A) Supplemental Figure: Proportions of mild, moderate, and severe substance use disorders (SUDs) among US adults with a specific SUD in the past year

B) Supplemental Table 1. Past-year prevalence of substance use, misuse, and use disorders among adults in the US overall and by age and sex (N=92,233)

C) Supplemental Table 2. Prevalence of specific combinations of substance use disorders among adults in the US overall and by age and sex (N=92,233)

D) Supplemental Table 3. Results of 3 multivariable Poisson regression models: Sociodemographic characteristics, health status, mental health conditions, and age initiation of specific substance use associated with (1) the number of different substances used/misused in the past year (secondary outcome); (2) the number of substance use disorders (SUDs) (primary outcome); (3) the number of moderate-severe SUDs (secondary outcome) among US adults

Supplemental Figure. Proportions of mild, moderate, and severe substance use disorders (SUDs) among US adults with a specific SUD in the past year, by SUD

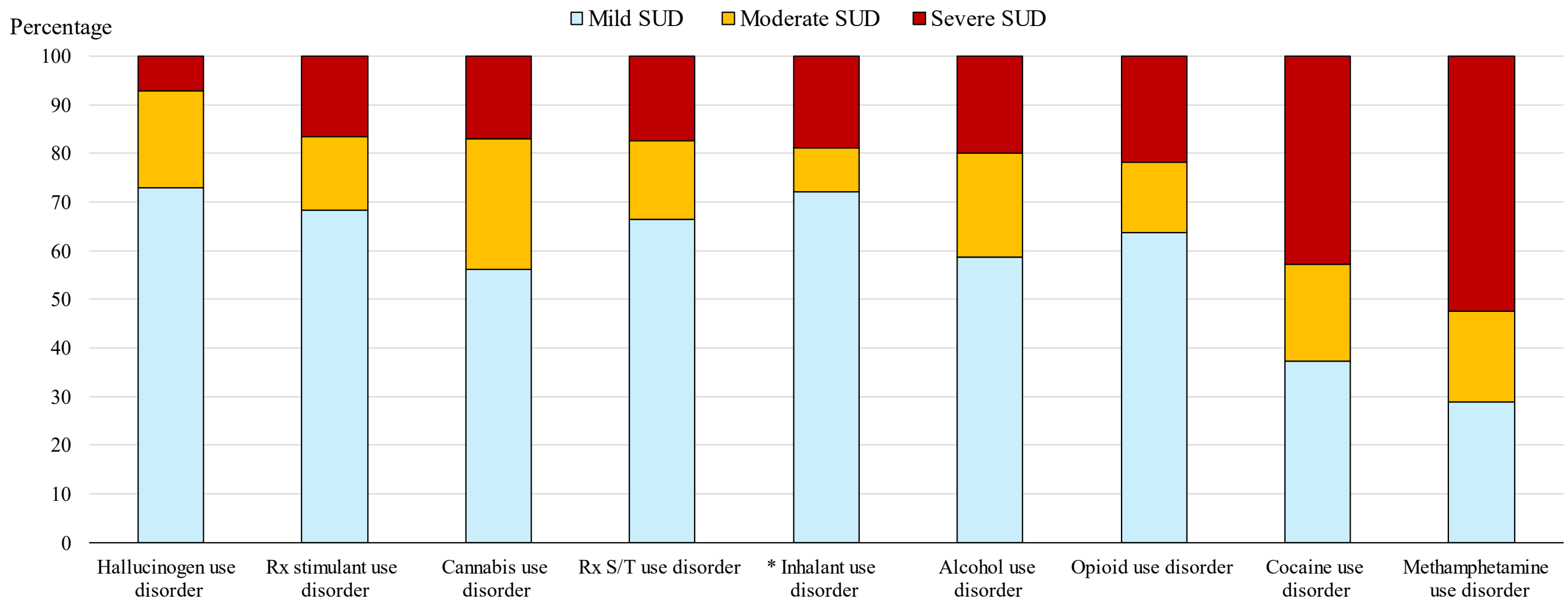

Source: 2022-2023 NSDUH data. S/T=sedative/tranquilizer. * Caution is needed when interpreting estimates due to low statistical precision.

Supplemental Table 1. Past-year prevalence [a] of substance use, misuse, and use disorders among adults in the US overall and by age and sex (N=92,233)

Substance Use or Substance Use Disorders	Weighted % (95% CI)				Weighted % (95% CI)	
	Ages 18+ N=92,233	Ages 18-29 n=35,781	Ages 30-49 n=35,872	Ages 50+ n=20,580	Males n=41,025	Females n=51,208
**Substance Use**						
Alcohol	67.2 (66.5-67.8)	71.0 (70.1-71.8)	72.6 (71.9-73.4)	61.5 (60.5-62.6)	69.3 (68.6-70.0)	65.1 (64.2-66.1)
Tobacco products or nicotine vaping	29.2 (28.5-29.8)	40.3 (39.3-41.4)	34.5 (33.4-35.6)	20.5 (19.6-21.4)	35.4 (34.5-36.3)	23.3 (22.5-24.1)
Cannabis	23.0 (22.5-23.4)	37.5 (36.6-38.4)	27.3 (26.4-28.2)	13.5 (12.9-14.2)	25.8 (25.0-26.5)	20.3 (19.7-20.9)
Rx opioids (no opioid misuse)	24.4 (23.8-25.0)	16.3 (15.6-17.1)	22.6 (21.9-23.2)	29.2 (28.3-30.1)	22.1 (21.2-23.1)	26.5 (25.8-27.3)
Opioid misuse	3.3 (3.1-3.6)	3.2 (2.9-3.5)	4.5 (4.1-4.8)	2.6 (2.3-3.0)	3.6 (3.3-4.0)	3.1 (2.9-3.3)
Rx S/T (no misuse)	13.4 (13.0-13.9)	7.3 (6.9-7.8)	12.4 (11.8-13.0)	16.8 (16.1-17.6)	10.0 (9.3-10.8)	16.7 (16.1-17.2)
Rx S/T misuse	1.8 (1.7-2.0)	2.0 (1.8-2.3)	2.4 (2.2-2.6)	1.3 (1.1-1.6)	1.7 (1.5-2.0)	1.9 (1.7-2.1)
Rx stimulants (no misuse)	5.4 (5.1-5.7)	7.4 (6.9-7.9)	7.0 (6.6-7.5)	3.3 (3.0-3.8)	4.9 (4.5-5.3)	5.9 (5.6-6.2)
Rx stimulant misuse	1.5 (1.4-1.6)	3.6 (3.2-4.0)	1.9 (1.7-2.1)	0.3 (0.2-0.4)	1.7 (1.5-1.9)	1.3 (1.2-1.5)
Hallucinogens	3.2 (3.1-3.4)	7.4 (7.0-7.9)	4.1 (3.8-4.4)	0.8 (0.6-1.0)	4.0 (3.7-4.2)	2.5 (2.3-2.7)
Cocaine	2.0 (1.8-2.2)	3.8 (3.4-4.2)	2.3 (2.1-2.7)	0.9 (0.8-1.2)	2.7 (2.4-3.0)	1.3 (1.1-1.5)
Methamphetamine	1.0 (0.9-1.2)	0.6 (0.5-0.9)	1.7 (1.5-2.0)	0.7 (0.5-0.9)	1.4 (1.1-1.6)	0.7 (0.6-0.9)
Inhalants	0.7 (0.6-0.8)	1.9 (1.6-2.2)	0.8 (0.7-1.0)	0.2 (0.1-0.3)	1.1 (0.9-1.3)	0.4 (0.3-0.5)
**Substance Use Disorders**						
Alcohol use disorder	11.1 (10.8-11.4)	16.1 (15.2-16.9)	13.6 (12.9-14.2)	7.1 (6.6-7.6)	13.5 (13.0-14.0)	8.8 (8.4-9.2)
Nicotine dependence	9.3 (8.9-9.7)	5.3 (4.9-5.7)	12.2 (11.5-12.9)	9.1 (8.5-9.6)	10.5 (9.9-11.0)	8.3 (7.8-8.8)
Cannabis use disorder	7.0 (6.7-7.2)	15.6 (15.1-16.1)	8.0 (7.5-8.5)	2.4 (2.1-2.8)	8.6 (8.1-9.0)	5.4 (5.1-5.7)
Opioid use disorder	2.2 (2.0-2.4)	1.4 (1.2-1.7)	2.4 (2.1-2.7)	2.4 (2.1-2.7)	2.2 (1.9-2.5)	2.2 (2.0-2.5)
Rx stimulant use disorder	0.6 (0.5-0.7)	1.2 (1.0-1.4)	0.8 (0.6-0.9)	0.2 (0.1-0.3)	0.6 (0.5-0.8)	0.5 (0.4-0.6)
Rx S/T use disorder	0.9 (0.8-1.0)	0.8 (0.7-1.0)	1.0 (0.9-1.2)	0.7 (0.6-0.9)	0.7 (0.6-0.9)	1.0 (0.8-1.2)
Hallucinogen use disorder	0.2 (0.1-0.2)	0.5 (0.3-0.6)	0.2 (0.1-0.3)	*	0.2 (0.2-0.3)	0.1 (0.1-0.2)
Cocaine use disorder	0.5 (0.4-0.6)	0.7 (0.6-1.0)	0.6 (0.5-0.8)	0.3 (0.2-0.4)	0.6 (0.5-0.8)	0.4 (0.3-0.4)
Methamphetamine use disorder	0.7 (0.6-0.8)	0.4 (0.3-0.6)	1.3 (1.1-1.6)	0.4 (0.3-0.6)	0.9 (0.8-1.1)	0.5 (0.4-0.7)
Inhalant use disorder	0.1 (0.1-0.1)	0.2 (0.1-0.3)	0.1 (0.1-0.2)	*	0.1 (0.1-0.2)	0.0 (0.0-0.1)

Data sources: 2022-2023 National Surveys on Drug Use and Health. Rx=prescription; S/T=sedative/tranquilizer; Opioid misuse= misuse of Rx opioids or use of heroin or illegally made fentanyl. [a]: All outcomes presented in this table are past-year measures, except for past-month nicotine dependence. *: Estimate suppressed due to low statistical precision.

Supplemental Table 2. Prevalence of specific combinations of substance use disorders [a] among adults in the US overall and by age and sex (N=92,233)

Substance Use Disorders	Weighted % (95% CI)				Weighted % (95% CI)	
	Ages 18+ N=92,233	Ages 18-29 n=35,781	Ages 30-49 n=35,872	Ages 50+ n=20,580	Males n=41,025	Females n=51,208
**Zero disorder**	76.2 (75.7-76.6)	70.3 (69.3-71.2)	71.8 (70.8-72.8)	81.9 (81.1-82.6)	72.5 (71.9-73.1)	79.6 (79.0-80.3)
**One disorder [b]**	17.6 (17.2-18.0)	20.6 (19.9-21.4)	19.9 (19.2-20.8)	14.6 (13.9-15.2)	19.9 (19.3-20.4)	15.5 (15.0-16.0)
AUD	7.1 (6.8-7.4)	9.1 (8.5-9.7)	8.6 (8.0-9.2)	5.2 (4.7-5.7)	8.5 (8.1-9.0)	5.7 (5.4-6.0)
ND	5.8 (5.5-6.1)	2.0 (1.8-2.3)	6.8 (6.4-7.3)	6.7 (6.2-7.2)	6.0 (5.6-6.5)	5.6 (5.2-6.0)
CUD	3.3 (3.1-3.5)	8.5 (8.0-8.9)	3.3 (3.0-3.6)	1.0 (0.8-1.3)	4.0 (3.7-4.3)	2.6 (2.4-2.8)
OUD	0.9 (0.7-1.0)	0.4 (0.3-0.6)	0.6 (0.5-0.8)	1.2 (1.0-1.5)	0.7 (0.6-0.9)	1.0 (0.8-1.1)
Rx S/TUD	0.2 (0.2-0.3)	0.2 (0.1-0.4)	0.2 (0.1-0.3)	0.3 (0.2-0.4)	0.1 (0.1-0.2)	0.3 (0.2-0.4)
MethUD	0.2 (0.1-0.2)	0.1 (0.0-0.1)	0.2 (0.1-0.4)	0.2 (0.1-0.3)	0.2 (0.1-0.4)	0.1 (0.0-0.2)
Rx StUD	0.1 (0.1-0.2)	0.3 (0.2-0.4)	0.2 (0.2-0.3)	*	0.2 (0.1-0.2)	0.1 (0.1-0.2)
**Two disorders [b]**	4.6 (4.4-4.9)	6.8 (6.4-7.2)	5.8 (5.4-6.3)	2.8 (2.5-3.3)	5.7 (5.3-6.0)	3.7 (3.3-4.0)
AUD + CUD	1.4 (1.3-1.6)	3.8 (3.5-4.1)	1.5 (1.3-1.7)	0.4 (0.2-0.6)	1.8 (1.6-2.0)	1.1 (1.0-1.3)
ND + AUD	1.1 (1.0-1.2)	0.8 (0.6-1.0)	1.5 (1.3-1.8)	0.9 (0.7-1.1)	1.4 (1.2-1.6)	0.8 (0.7-0.9)
ND + CUD	0.9 (0.7-1.0)	1.0 (0.8-1.2)	1.3 (1.1-1.5)	0.5 (0.3-0.7)	1.1 (1.0-1.3)	0.6 (0.5-0.7)
ND + OUD	0.2 (0.2-0.3)	0.1 (0.0-0.1)	0.3 (0.2-0.4)	0.3 (0.2-0.4)	0.2 (0.2-0.3)	0.3 (0.2-0.3)
AUD + OUD	0.1 (0.1-0.2)	0.1 (0.0-0.3)	0.1 (0.1-0.2)	0.2 (0.1-0.3)	0.1 (0.1-0.2)	0.1 (0.1-0.2)
ND + MethUD	0.1 (0.1-0.1)	0.0 (0.0-0.0)	0.2 (0.2-0.3)	0.0 (0.0-0.1)	0.2 (0.1-0.2)	0.1 (0.0-0.1)
CUD + OUD	0.1 (0.1-0.2)	0.1 (0.1-0.2)	0.1 (0.1-0.2)	0.1 (0.0-0.2)	0.1 (0.1-0.3)	0.1 (0.0-0.1)
AUD + CocUD	0.1 (0.1-0.1)	0.1 (0.1-0.2)	0.1 (0.1-0.3)	*	0.1 (0.0-0.2)	0.1 (0.1-0.1)
OUD + Rx S/TUD	0.1 (0.1-0.1)	0.0 (0.0-0.1)	0.1 (0.0-0.2)	0.1 (0.1-0.2)	0.0 (0.0-0.1)	0.1 (0.1-0.2)
ND + Rx S/TUD	0.1 (0.0-0.1)	0.0 (0.0-0.1)	*	0.1 (0.1-0.2)	0.1 (0.0-0.2)	0.1 (0.0-0.1)
**≥Three disorders [b, c]**	1.6 (1.5-1.8)	2.3 (2.0-2.6)	2.4 (2.1-2.7)	0.7 (0.6-0.9)	2.0 (1.7-2.3)	1.3 (1.1-1.4)
ND + AUD + CUD	0.4 (0.3-0.5)	0.6 (0.4-0.7)	0.6 (0.5-0.8)	0.1 (0.1-0.2)	0.5 (0.4-0.6)	0.3 (0.2-0.3)
ND + CUD + OUD	0.1 (0.0-0.1)	0.1 (0.0-0.1)	0.1 (0.1-0.2)	*	0.1 (0.1-0.1)	0.1 (0.0-0.1)

Data sources: 2022-2023 National Surveys on Drug Use and Health. ND=nicotine dependence; AUD=alcohol use disorder; CUD=cannabis use disorder; Rx=prescription; OUD=opioid use disorder; S/TUD=sedative or tranquilizer use disorder; StUD=stimulant use disorder. MethUD=methamphetamine use disorder; CocUD=cocaine use disorder.

[a]:Substance use disorders examined in this analysis include past-year alcohol use disorder, cannabis use disorder, opioid use disorder, Rx stimulant use disorder, Rx sedative or tranquilizer use disorder, cocaine use disorder, hallucinogen use disorder, methamphetamine use disorder, and inhalant use disorder as well as past-month nicotine dependence.

There were ***299 unique, exclusive combinations*** of aforementioned substance use disorders among adults aged ≥18. [b]:Among adults aged ≥18, individual substance use disorders or combinations of substance use disorders were not presented if prevalence was <0.1% due to limited space. [c]: Individual combinations of disorders with 4 or more disorders were not presented due to low prevalence (each combination: <0.1%).

*: Estimate suppressed due to low statistical precision.

Supplemental Table 3. Results of 3 multivariable Poisson regression models: Sociodemographic characteristics, health status, mental health conditions, and age initiation of specific substance use associated with (1) the number of different substances used/misused in the past year (secondary outcome); (2) the number of substance use disorders (SUDs) (primary outcome); (3) the number of moderate-severe SUDs (secondary outcome) among US adults

Sociodemographic Characteristics, Health Status, and Behavioral Health Conditions	Model 4 outcome: Number of different substances used/misused [c] Incidence Density Ratio (95% CI) (N=92,233)	Model 5 outcome: Number of SUDs [d] Incidence Density Ratio (95% CI) (N=92,233)	Model 6 outcome: Number of moderate-severe alcohol/drug use disorders [e] Incidence Density Ratio (95% CI) (N=92,233)
Sociodemographic Characteristics			
Age			
18-29 [a]	1.00	1.00	1.00
30-49	**0.88 (0.87-0.90)**	**0.90 (0.85-0.95)**	**0.66 (0.61-0.71)**
≥50	**0.76 (0.74-0.77)**	**0.61 (0.56-0.66)**	**0.33 (0.29-0.39)**
Sex			
Male	**0.97 (0.95-0.98)**	**1.12 (1.09-1.16)**	**1.26 (1.16-1.39)**
Female [a]	1.00	1.00	1.00
Race/ethnicity			
Hispanic	**0.94 (0.92-0.96)**	**0.90 (0.85-0.95)**	1.02 (0.92-1.13)
NH adult with >1 race	1.00 (0.95-1.04)	1.04 (0.96-1.12)	0.96 (0.84-1.09)
NH American Indian or Alaska Native	1.04 (0.97-1.11)	1.18 (1.00-1.39)	**1.59 (1.16-2.18)**
NH Asian	**0.90 (0.86-0.94)**	0.97 (0.83-1.13)	0.99 (0.80-1.23)
NH Black	**1.04 (1.02-1.06)**	**1.19 (1.12-1.25)**	**1.40 (1.27-1.55)**
NH NHOPI	0.95 (0.85-1.06)	1.25 (0.91-1.73)	1.09 (0.48-2.49)
NH White [a]	1.00	1.00	1.00
Education attainment			
<High school		**1.55 (1.44-1.67)**	**1.37 (1.19-1.56)**
High school		**1.30 (1.24-1.37)**	1.11 (1.00-1.24)
Some college or associate degree		**1.14 (1.09-1.20)**	**1.13 (1.03-1.23)**
College graduate [a]		1.00	1.00
Family income			
<$20,000		**1.28 (1.19-1.37)**	**1.16 (1.05-1.28)**
$20,000-$49,999		**1.14 (1.08-1.21)**	1.06 (0.98-1.14)
$50,000-$74,999		**1.10 (1.04-1.16)**	1.12 (1.00-1.25)
≥$75,000 [a]		1.00	1.00
Employment status			
Full-time [a]	1.00	1.00	1.00
Part-time	1.00 (0.98-1.02)	0.99 (0.93-1.06)	1.07 (0.96-1.19)
Unemployed	**1.08 (1.04-1.11)**	**1.20 (1.11-1.29)**	**1.56 (1.36-1.79)**
Disabled	**1.10 (1.07-1.14)**	**1.11 (1.03-1.20)**	0.90 (0.74-1.08)
Other (retired; not looking for a job)	**0.97 (0.95-0.99)**	**0.94 (0.89-0.99)**	1.02 (0.92-1.15)
Marital status			
Married [a]	1.00	1.00	1.00
Divorced or separated	**1.06 (1.04-1.09)**	**1.29 (1.21-1.37)**	**1.42 (1.24-1.62)**
Widowed	1.01 (0.97-1.06)	1.16 (0.99-1.35)	1.08 (0.76-1.54)
Never married	**1.09 (1.07-1.11)**	**1.29 (1.23-1.35)**	**1.52 (1.39-1.67)**
Health insurance			
Private only [a]	1.00	1.00	
Uninsured	0.98 (0.95-1.00)	**1.12 (1.04-1.21)**	
Medicaid only	1.00 (0.98-1.03)	**1.08 (1.01-1.15)**	
Other (e.g., Medicare)	**0.96 (0.94-0.98)**	0.96 (0.91-1.03)	
Metropolitan statistical area			
Large			**1.13 (1.02-1.25)**
Small			1.09 (0.99-1.20)
Nonmetropolitan [a]			1.00
Health Status			
Self-rated health			
Excellent [a]	1.00	1.00	1.00
Very good	**1.08 (1.06-1.11)**	**1.17 (1.10-1.24)**	**1.19 (1.05-1.35)**
Good	**1.13 (1.11-1.16)**	**1.42 (1.33-1.51)**	**1.43 (1.27-1.62)**
Fair or poor	**1.19 (1.16-1.22)**	**1.60 (1.48-1.73)**	**1.73 (1.46-2.04)**
Emergency department visit			
0[a]	1.00	1.00	1.00
1-2	**1.13 (1.11-1.16)**	**1.14 (1.09-1.19)**	**1.27 (1.17-1.37)**
≥3	**1.22 (1.18-1.27)**	**1.21 (1.11-1.33)**	**1.32 (1.14-1.54)**
Mental Health Conditions			
Suicidal ideation			
Yes	**1.10 (1.07-1.14)**	**1.25 (1.19-1.32)**	**1.46 (1.34-1.58)**
No [a]	1.00	1.00	1.00

Supplemental Table 3. (Con't)

Sociodemographic Characteristics, Health Status, and Behavioral Health Conditions	Outcome: Number of types of substances used/misused<br>Incidence Density Ratio (IDR) 95% CI)	Outcome: Number of SUDs<br>IDR (95% CI)	Outcome: Number of moderate-severe alcohol/drug use disorders<br>IDR (95% CI)
Major depressive episode			
Yes	**1.07 (1.05-1.08)**	**1.28 (1.21-1.36)**	**1.56 (1.44-1.69)**
No [a]	1.00	1.00	1.00
Age of First Substance Use by Substance [b]			
Alcohol			
≤17 [a]	1.00	1.00	1.00
18-20	1.00 (0.99-1.02)	**0.93 (0.89-0.97)**	**0.89 (0.74-0.99)**
21-29	**0.95 (0.93-0.97)**	**0.84 (0.77-0.92)**	**0.68 (0.57-0.83)**
≥30	0.93 (0.86-1.01)	0.91 (0.69-1.19)	0.79 (0.41-1.53)
Never use	**0.36 (0.34-0.38)**	**0.71 (0.62-0.81)**	**0.55 (0.40-0.76)**
Tobacco products or nicotine vaping			
≤17 [a]	1.00	1.00	1.00
18-20	0.98 (0.96-1.00)	0.96 (0.90-1.02)	1.01 (0.93-1.11)
21-29	0.99 (0.97-1.02)	0.95 (0.87-1.03)	1.02 (0.89-1.17)
≥30	**1.10 (1.03-1.18)**	0.80 (0.62-1.04)	1.05 (0.76-1.46)
Never use	**0.72 (0.71-0.73)**	**0.35 (0.31-0.38)**	**0.45 (0.36-0.55)**
Cannabis			
≤17 [a]	1.00	1.00	1.00
18-20	**0.96 (0.94-0.98)**	**0.88 (0.83-0.93)**	**0.84 (0.76-0.93)**
21-29	**0.96 (0.93-0.99)**	**0.86 (0.79-0.93)**	**0.79 (0.69-0.90)**
≥30	**1.08 (1.03-1.13)**	**0.85 (0.76-0.95)**	0.91 (0.70-1.18)
Never use	**0.72 (0.70-0.74)**	**0.54 (0.50-0.58)**	**0.38 (0.31-0.45)**
Hallucinogens			
≤17 [a]	1.00	1.00	1.00
18-20	0.99 (0.95-1.03)	0.98 (0.91-1.06)	0.95 (0.86-1.05)
21-29	0.99 (0.96-1.02)	1.01 (0.94-1.09)	1.06 (0.92-1.25)
≥30	**1.13 (1.07-1.20)**	1.13 (0.96-1.32)	1.18 (0.88-1.59)
Never use	**0.83 (0.80-0.85)**	**0.74 (0.69-0.79)**	**0.65 (0.57-0.75)**
Cocaine			
≤17 [a]	1.00	1.00	1.00
18-20	0.99 (0.95-1.03)	1.04 (0.94-1.16)	1.12 (0.95-1.32)
21-29	0.99 (0.94-1.04)	0.99 (0.90-1.09)	1.03 (0.86-1.24)
≥30	0.95 (0.90-1.01)	1.01 (0.87-1.16)	0.95 (0.70-1.29)
Never use	**0.87 (0.84-0.91)**	**0.76 (0.69-0.84)**	**0.64 (0.54-0.75)**
Methamphetamine			
≤17 [a]	1.00	1.00	1.00
18-20	0.96 (0.90-1.02)	**0.89 (0.80-0.99)**	0.92 (0.72-1.19)
21-29	1.01 (0.94-1.08)	0.97 (0.84-1.11)	0.95 (0.76-1.18)
≥30	**1.12 (1.05-1.19)**	1.07 (0.93-1.23)	0.97 (0.73-1.28)
Never use	**0.94 (0.90-0.98)**	**0.83 (0.76-0.91)**	**0.77 (0.64-0.93)**
Heroin			
≤17 [a]		1.00	1.00
18-20		0.95 (0.75-1.20)	0.81 (0.60-1.09)
21-29		0.96 (0.74-1.25)	0.87 (0.65-1.15)
≥30		1.11 (0.83-1.49)	1.24 (0.83-1.86)
Never use		0.82 (0.65-1.03)	**0.68 (0.52-0.89)**
Inhalants			
≤17 [a]	1.00	1.00	1.00
18-20	**1.05 (1.003-1.10)**	1.00 (0.90-1.11)	1.06 (0.88-1.28)
21-29	**1.10 (1.05-1.15)**	1.08 (0.96-1.23)	0.99 (0.82-1.20)
≥30	**1.20 (1.09-1.33)**	1.14 (0.90-1.44)	1.23 (0.88-1.72)
Never use	**0.95 (0.92-0.97)**	**0.88 (0.83-0.93)**	**0.75 (0.67-0.85)**

Data source: 2022-2023 National Surveys on Drug Use and Health. NH=Non-Hispanic; NHOPI=Native Hawaiian/Other Pacific Islander. [a]: Reference group. [b]: Age of first misuse was unavailable for non-past-year initiates of prescription medication misuse. **Bold** prevalence was statistically significantly different from the estimate of the reference group within each table cell (*P*<.05). [c]: The final Poisson regression model for the number of substances used/misused in the past year: Education attainment, family income, metropolitan statistical area (MSA), and age of first heroin use were not associated with the outcome and were excluded from the final model. [d]: The final model for the number of SUDs: MSA was not associated with the outcome and was excluded from the final model. [e]: The final model for the number of moderate-severe SUDs: Health insurance was not associated with the outcome and was excluded from the final model.
